# The Association between Five Genetic Variants in MicroRNAs (rs2910164, rs11614913, rs3746444, rs11134527, and rs531564) and Cervical Cancer Risk: A Meta-Analysis

**DOI:** 10.1155/2021/9180874

**Published:** 2021-03-15

**Authors:** Jia Liu, Peng Dong, Liane Zhou, Shijun Wang

**Affiliations:** Department of Obstetrics and Gynecology, Xuanwu Hospital, Capital Medical University, Beijing, China

## Abstract

The objective of this study was to conduct a meta-analysis to systematically summarize and investigate the association of miRNA-124 rs531564, miRNA-218 rs11134527, miRNA-146a rs2910164, miRNA-196a2 rs11614913, and miRNA-499 rs3746444 polymorphisms with cervical cancer. A systematic review was performed to identify relevant studies using Embase and PubMed databases. A chi-square-based *Q*-test combined with the inconsistency index (*I*^2^) was used to check the heterogeneity between studies. A total of six case-control studies on rs2910164 and rs11614913, 4 studies on rs3746444 and rs11134527, and three studies on rs531564 were included. No evidence of association was found between miR-146a rs2910164, miR-196a2 rs11614913, miRNA-499 rs3746444, and miR-218 rs11134527 polymorphisms and cervical cancer risk in all the genetic models. The miR-124 rs531564 polymorphism was associated with a statistically increased risk of cervical cancer in a homozygote model (CC vs. GG: OR = 2.87, 95% CI: 1.40-5.91, *P*_*H*_ = 0.887), dominant model (GC/CC vs. GG: OR = 1.38, 95% CI: 1.07-1.80, *P*_*H*_ = 0.409), and recessive model (CC vs. GC/GG: OR = 2.26, 95% CI: 1.58-3.23, *P*_*H*_ = 0.979). However, this finding should be interpreted with caution for limited samples and heterogeneity. Large-scale and well-designed studies are needed to validate our result.

## 1. Introduction

Cervical cancer (CC) is the fourth most common cancer in females, with 570,000 new cases and 311,000 deaths estimated for 2018 worldwide [1]. Cervical cancer is a multistep process involving the transformation of the normal cervical epithelium to cervical intraepithelial neoplasia that is subsequently transformed to cervical cancer [2]. Due to cervical cytology screening, cervical cancer can be detected at an early stage, greatly reducing the incidence and mortality. However, cervical cancer is still one of the deadliest female-specific cancers due to its tendency to metastasize and recur after treatment. It is well established that the persistence of Human Papillomavirus (HPV) infection is the main cause of cervical cancer and is indeed deemed as a necessary cause for the disease. However, evidence suggests that genetic risk factors also play a crucial role in the pathogenesis of cervical cancer [3–5].

MicroRNAs (miRNAs) are short nonprotein-coding small RNAs of approximately 23 nucleotides that mediate posttranscriptional regulation through base pairing to partially complementary sites of the target mRNA and promote translational repression or messenger RNA degradation [6, 7]. Increasing evidences show that single nucleotide polymorphisms (SNPs) or variation in miRNA sequence could potentially alter various biological processes by influencing target selection of miRNAs and is closely associated with various human diseases [8]. The relationship between genetic mutations in microRNA and cancer susceptibility has been getting increasing attention. Several recent studies have indicated that miRNA-related SNPs can remarkably alter the biogenesis and function of the corresponding miRNAs and may function as oncogenic or antioncogenic molecules in human cancers [9, 10].

Recently, though a number of studies have been conducted to investigate the association of miR-124 G>C (rs531564), miR-218 A>G (rs11134527), miR-146a G>C (rs2910164), miR-196a2 T>C (rs11614913), and miR-499 A>G (rs3746444) polymorphisms with cervical cancer [2, 11–20], the results were inconclusive. Therefore, a meta-analysis was performed to evaluate the association between these five SNPs and the risk of cervical cancer.

## 2. Methods and Materials

### 2.1. Publication Search

The PubMed and Embase databases were searched by two independent investigators covering all papers published up to December 27, 2020. The following terms were used: “cervical or CIN”, “microRNA or miRNA or microRNAs”, and “polymorphism or polymorphisms”. The references of the retrieved articles and review articles were also searched manually for further relevant studies. All eligible studies met the following criteria: (1) full-text study, (2) case-control design, (3) analysis of the relationship between miRNA polymorphisms and the risk of cervical cancer, (4) studies focusing on human beings, and (5) sufficient data to calculate risk estimates: the odds ratio (OR) with 95% confidence interval (CI) and a *P* value. The selection of the study was completed independently by two investigators (Liu and Dong) according to the inclusion criteria.

### 2.2. Data Extraction

Each publication was sought for the following information: the first author's surname, the publication year, country of origin, ethnicity, genotyping method, the number of cases and controls, and Hardy-Weinberg equilibrium (HWE). According to the amount of the sample, we defined sample size as the large sample size (≥200) and small sample size (<200).

Two reviewers (Liu and Dong) extracted the eligible research data repeatedly. Disagreement between two reviewers was discussed with another reviewer (Wang) until a consensus is reached.

When Hardy-Weinberg equilibrium (HWE) in the control was not reported, the *χ*^2^ test was used to evaluate the HWE of polymorphism in the control group. If *P* < 0.05, it is considered deviated from HWE.

### 2.3. Methodological Quality Assessment

The quality of the selected studies was assessed according to the methodological quality assessment scale which was mentioned in prior meta-analysis [22] (see Table S1).

In this scale, five items were evaluated, including (1) the representativeness of the subjects, (2) the ascertainment of cervical cancer, (3) the control of the genotyping methods, (4) HWE in controls, and (5) assessment of association. The quality score ranged from 0 to 9, and a high score indicated good quality of the study. Studies with a score less than 5 were removed from the subsequent analyses.

### 2.4. Statistics

We conducted our meta-analysis based on the PRISMA checklists and followed the guideline [23]. The strength of association was assessed between miRNA polymorphisms and the risk of cervical cancer by crude OR corresponding to 95% CI [24]. Pooled ORs were performed for allelic comparison (miR-146a: G versus C, miR-196a: T versus C, miR-499: A versus G, miR-218: A versus G, and miR-124: G versus C), heterozygote model (miR-146a: GC versus GG, miR-196a: TC versus TT, miR-499: AG versus AA, miR-218: AG versus AA, and miR-124: GC versus GG), homozygote model (miR-146a: CC versus GG, miR-196a: CC versus TT, miR-499: GG versus AA, miR-218: GG versus AA, and miR-124: CC versus GG), dominant model (miR-146a: CC+GC versus GG, miR-196a: TC+CC versus TT, miR-499: AG+GG versus AA, miR-218: AG+GG versus AA, and miR-124: GC+CC versus GG), and recessive model (miR-146a: CC versus CG+GG, miR-196a: CC versus TC+TT, miR-499: GG versus AG+AA, miR-218: GG versus AG+AA, and miR-124: CC versus GC+GG), respectively.

A chi-square-based *Q*-test combined with the inconsistency index (*I*^2^) was used to check the heterogeneity between studies [25]. The random-effects model was used when heterogeneity tests yielded significant results (*P* < 0.1 or *I*^2^ ≥ 50%) [26, 27]. Besides, subgroup analyses were stratified by sample size and HWE. Publication bias was assessed by visual inspection of funnel plots and using Egger's test. Sensitivity analyses were performed to identify individual studies' effect on pooled results and test the reliability of results. *P* value of Egger's test <0.05 was considered representative of statistically significant publication bias. All statistical analyses were carried out using the STATA 12.0 software (Stata Corp., College Station, TX, USA).

## 3. Results

### 3.1. Study Characteristics

**Figure 1:**
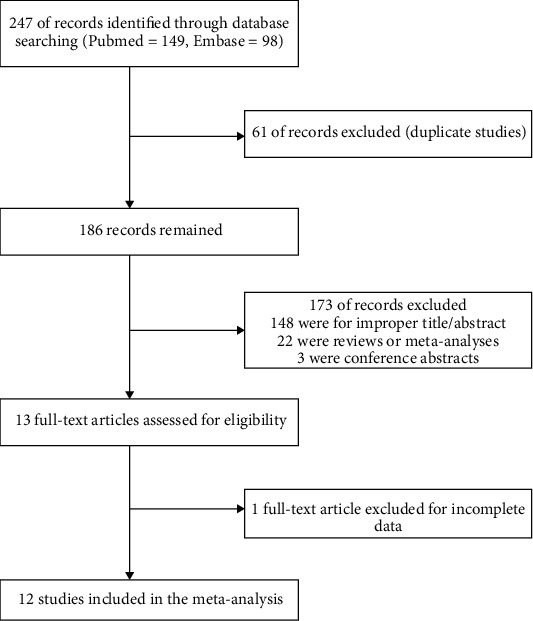
Flow chart of study selection.

A total of 247 studies were acquired from Embase and PubMed databases (Embase: 98 and PubMed: 149). The detailed screening process is shown in Figure 1. After reading the titles and the abstracts, 235 articles were excluded, of which 61 were duplicate ones, 149 had no relation to this topic, 22 were reviews or meta-analyses, and 3 were conference abstracts. Finally, 12 eligible case-control studies were included in our meta-analysis (Table 1) [10–21].

In six studies, genotype frequencies of rs531564, rs11134527, rs2910164, rs11614913, and rs3746444 were presented separately, so each of them was considered as a separate study in this meta-analysis. Therefore, all the six included studies containing 2291 cases and 2850 controls for rs2910164, six studies containing 2291 cases and 2850 controls for rs11614913, four studies involving 1554 cases and 1962 controls for rs3746444, four studies containing 3167 cases and 3132 controls for rs11134527, and three studies containing 975 cases and 950 controls for rs531564 were finally analyzed in our meta-analysis. For these five miRNA in this meta-analysis, the subjects in all included studies were of the Asian population.

The TaqMan assay and polymerase chain reaction-restriction fragment length polymorphism (PCR-RFLP) were used for genotyping to determine the SNPs in the included studies. HWE of genotype distribution in the controls was tested in all studies, and two articles were not consistent with HWE. And according to the methodology quality assessment, all the studies were above a score of 6.0 and recruited into the following analyses.

### 3.2. Association between miRNA-146a rs2910164 Polymorphism and Cervical Cancer Susceptibility

**Figure 2:**
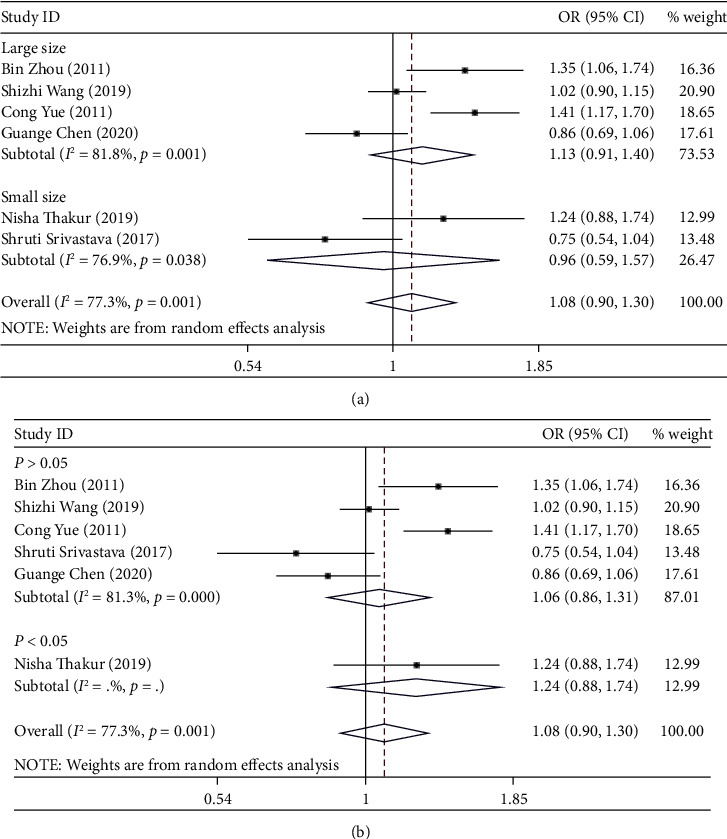
(a) Forest plot of allele comparison of miRNA-146a rs2910164 for overall comparison (C versus G). (b) Forest plot of allele comparison of miRNA-146a rs2910164 for overall comparison (C versus G).

The association strength between miRNA-146a rs2910164 polymorphism and cervical cancer risk is shown in Table 2. As shown in Table 2, significant association was not identified in any genetic model (C vs. G: OR = 1.08, 95% CI 0.90-1.30, *P*_*H*_ = 0.001, Figure 2; GC vs. GG: OR = 0.94, 95% CI 0.76-1.15, *P*_*H*_ = 0.140; CC vs. GG: OR =0.85, 95% CI 0.57-1.26, *P*_*H*_ = 0.001; GC/GG vs. CC: OR = 0.90, 95% CI 0.70-1.15, *P*_*H*_ = 0.014; and GG vs. GC/CC: OR = 0.90, 95% CI 0.68-1.19, *P*_*H*_ = 0.002).

Next, subgroup analysis was conducted according to sample size and HWE. In the large sample size and HWE group, a significant association was also not identified in any genetic model. And the heterogeneity is still high. When we delete three articles (Shizhi Wan, Shruti SrivaStava, and Guange Chen), heterogeneity is significantly reduced (Table 3). A significantly increased risk of cervical cancer susceptibility was identified in allelic comparison (C vs. G: OR = 1.36, 95% CI: 1.19-1.56, *P*_*H*_ = 0.800), while there is a significantly decreased risk of cervical cancer susceptibility in heterozygote comparison (GC vs. GG: OR = 0.76, 95% CI: 0.60-0.97, *P*_*H*_ = 0.379), homozygote model (CC vs. GG: OR = 0.53, 95% CI: 0.40-0.70, *P*_*H*_ = 0.683), dominant model (GC/CC vs. GG: OR = 0.68, 95% CI: 0.54-0.85, *P*_*H*_ = 0.406), and recessive model (CC vs. GC/GG: OR = 0.66, 95% CI: 0.53-0.82, *P*_*H*_ = 0.638).

### 3.3. Association between miR-196a2 rs11614913 Polymorphism and Cervical Cancer Susceptibility

**Figure 3:**
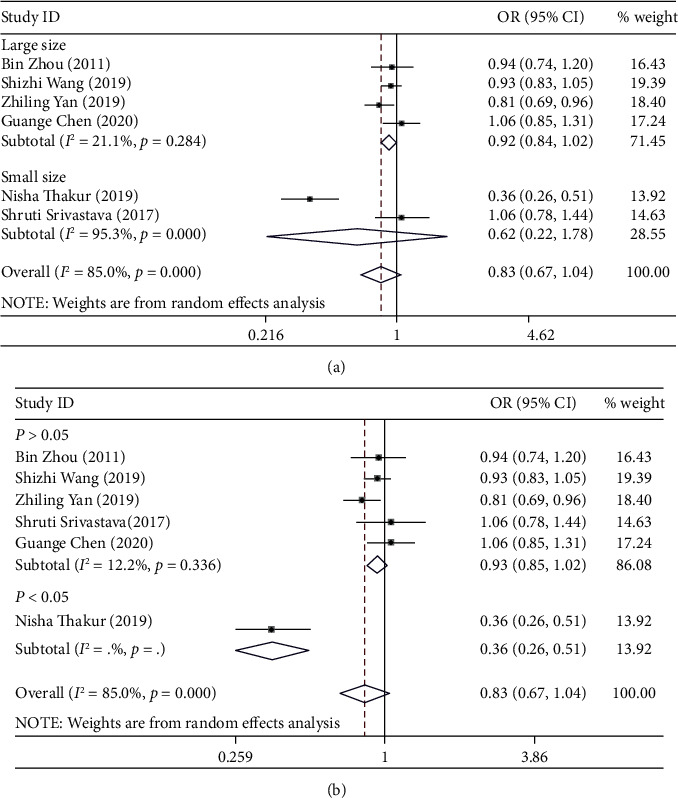
(a) Forest plot of allele comparison of miRNA-196a2 rs11614913 for overall comparison (T versus C). (b) Forest plot of allele comparison of miRNA-196a2 rs11614913 for overall comparison (T versus C).

Significant association was not identified in any genetic model (T vs. C: OR = 0.83, 95% CI: 0.67-1.04, *P*_*H*_ = 0.000, Figure 3; TC vs. TT: OR = 1.19, 95% CI: 0.90-1.58, *P*_*H*_ = 0.001; CC vs. TT: OR = 141, 95% CI: 0.94-2.10, *P*_*H*_ = 0.000; TC/CC vs. TT: OR = 1.28, 95% CI: 0.93-1.75, *P*_*H*_ = 0.000; and CC vs. TC/TT: OR = 1.23, 95% CI: 0.97-1.29, *P*_*H*_ = 0.021) when all eligible studies were pooled.

Next, subgroup analysis was conducted according to sample size and HWE. In large sample size, heterogeneity is significantly reduced. A significantly increased risk of cervical cancer susceptibility was identified in homozygote comparison (CC vs. TT: OR = 1.19, 95% CI: 1.00-1.41, *P*_*H*_ = 0.369). In the HWE group, heterogeneity is also significantly reduced. But significant association was not identified in any genetic model.

### 3.4. Association between miR-499 rs3746444 Polymorphism and Cervical Cancer Susceptibility

**Figure 4:**
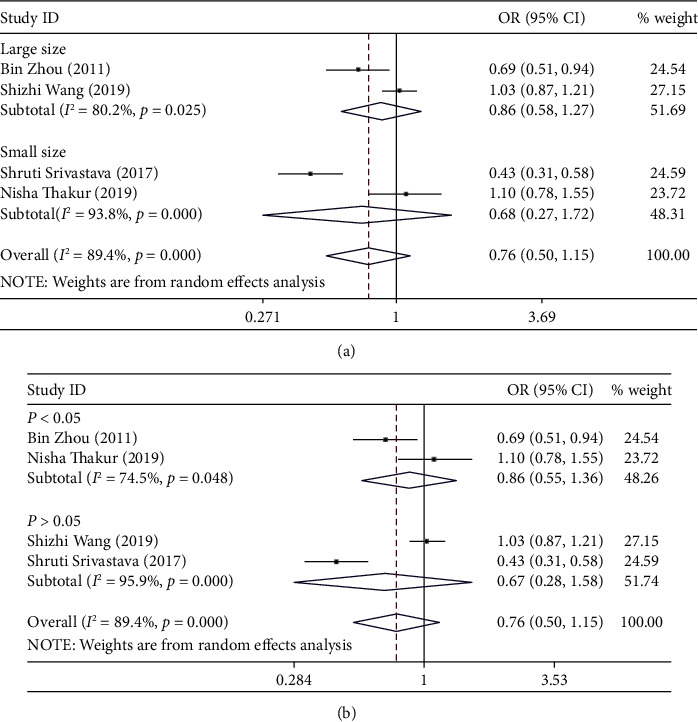
(a) Forest plot of allele comparison of miRNA-499 rs3746444 for overall comparison (G versus A). (b) Forest plot of allele comparison of miRNA-499 rs3746444 for overall comparison (G versus A).

Significant association was not identified in any genetic model (G vs. A: OR = 0.76, 95% CI: 0.50-1.15, *P*_*H*_ = 0.000, Figure 4; AG vs. AA: OR = 1.34, 95% CI: 0.82-2.20, *P*_*H*_ = 0.001; GG versus AA: OR = 1.42, 95% CI: 0.61-3.30, *P*_*H*_ = 0.000; AG/GG vs. AA: OR = 1.42, 95% CI: 0.83-2.42, *P*_*H*_ = 0.000; and GG vs. AG/AA: OR = 1.27, 95% CI: 0.68-2.35; *P*_*H*_ = 0.002).

Next, subgroup analysis was conducted according to sample size and HWE. In the large sample size and HWE group, a significant association was also not identified in any genetic model. The heterogeneity in the HWE group is still high, and in a large sample size, the heterogeneity is high except the homozygote model and recessive model. When we delete the two articles (Bin Zhou and Shruti SrivaStava), heterogeneity is significantly reduced in all models (Table 3). However, significant association was also not identified in any genetic model.

### 3.5. Association between miR-218 rs11134527 Polymorphism and Cervical Cancer Susceptibility

**Figure 5:**
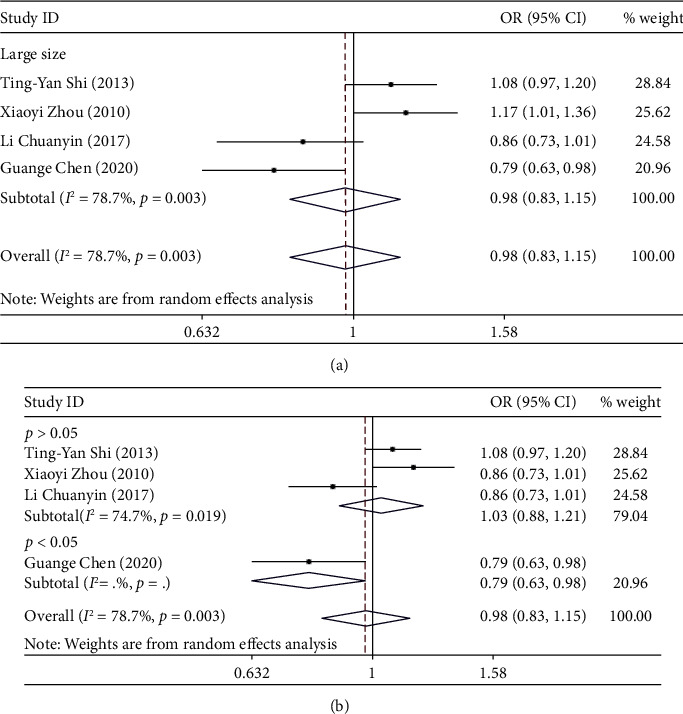
Forest plot of allele comparison of miR-218 rs11134527 for overall comparison (G versus A). (b) Forest plot of allele comparison of miR-218 rs11134527 for overall comparison (G versus A).

Significant association was not identified in any genetic model (G vs. A: OR = 0.98, 95% CI: 0.83-1.15, *P*_*H*_ = 0.003; AG vs. AA: OR = 1.07, 95% CI: 0.89-1.29, *P*_*H*_ = 0.053, Figure 5; GG vs. AA: OR = 1.01, 95% CI: 0.73-1.40, *P*_*H*_ = 0.006; AG/GG vs. AA: OR = 1.06, 95% CI: 0.86-1.31, *P*_*H*_ = 0.091; and GG vs. AG/AA: OR = 0.97, 95% CI: 0.76-1.23, *P*_*H*_ = 0.035).

The sample size of the 4 included articles is all large size and subgroup analysis was conducted according to HWE. In the HWE group, heterogeneity is also significantly reduced. But significant association was not identified in any genetic model. When we delete two articles (Li Chuanyin and Guange Chen), heterogeneity is significantly reduced in all models (Table 3). We observed a significantly increased risk of cervical cancer susceptibility in allelic comparison (G versus A: OR = 1.11, 95% CI: 1.02-1.21, *P*_*H*_ = 0.395), while there is a significantly decreased risk of cervical cancer susceptibility in the homozygote model (GG vs. AA: OR = 0.79, 95% CI: 0.66-0.94, *P*_*H*_ = 0.594) and recessive model (GG vs. AG/AA: OR = 0.80, 95% CI: 0.68-0.94, *P*_*H*_ = 0.982). Significant association was not identified in heterozygote comparison (AG vs. AA: OR = 0.97, 95% CI: 0.85-1.10; *P*_*H*_ = 0.214) and dominant model (GG/AG vs. AA: OR = 0.92, 95% CI: 0.81-1.04, *P*_*H*_ = 0.235).

### 3.6. Association between miR-124 rs531564 Polymorphism and Cervical Cancer Susceptibility

**Figure 6:**
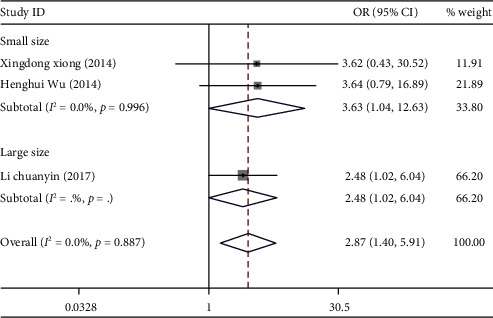
Forest plot of homozygote comparison of miR-124 rs531564 for overall comparison (CC versus GG).

We observed a significantly increased risk of cervical cancer susceptibility in the homozygote model (CC vs. GG: OR = 2.87, 95% CI: 1.40-5.91, *P*_*H*_ = 0.887, Figure 6), dominant model (GC/CC vs. GG: OR = 1.38, 95% CI: 1.07-1.80, *P*_*H*_ = 0.409), and recessive model (CC vs. GC/GG: OR = 2.26, 95% CI: 1.58-3.23, *P*_*H*_ = 0.979), while there is a significantly decreased risk of cervical cancer susceptibility in allelic comparison (C vs. G: OR = 0.58, 95% CI: 0.40-0.83, *P*_*H*_ = 0.097). Significant association was not identified in heterozygote comparison (GC vs.GG: OR = 1.27, 95% CI: 0.97-1.66, *P*_*H*_ = 0.899).

Next, subgroup analysis was conducted according to sample size. In a large sample size, a significantly increased risk of cervical cancer susceptibility could be identified in the homozygote model (CC vs. GG: OR = 3.63, 95% CI: 1.04-12.63, *P*_*H*_ = 0.998) and recessive model (CC vs. GC/GG: OR = 2.24, 95% CI: 1.51-3.31, *P*_*H*_ = 0.86), while there is a significantly decreased risk of cervical cancer susceptibility in allelic comparison (C vs. G: OR = 0.46, 95% CI: 0.32-0.66; *P*_*H*_ = 0.858). Significant association was not identified in heterozygote comparison (GC vs.GG: OR = 1.27, 95% CI: 0.97-1.66, *P*_*H*_ = 0.899) and dominant model (GC/CC vs. GG: OR = 3.13, 95% CI: 0.90-10.85, *P*_*H*_ = 0.995).

### 3.7. Sources of Heterogeneity

Since significant heterogeneity was found for miR-146 (rs2910164), miR-196a2 (rs11614913), miR-499 (rs3746444), and miR-218(rs11134527), we conducted a subgroup analysis by sample size, and the results showed that the sample size is the source of heterogeneity of miR-196a2 (rs11614913) in each genetic model. However, for other SNPs, heterogeneity was still high in large sample size. However, when we delete some articles, we found that the heterogeneity is significantly reduced.

### 3.8. Sensitivity Analysis

The influence of each study on the pooled ORs in this meta-analysis was examined by excluding one study at a time using STATA 12.0 software. The results showed that no significant alteration in the pooled ORs was found in any of the genetic models for the five SNPs.

### 3.9. Publication Bias

Due to the limited number of studies included (<10), no funnel plot or Egger's test was performed.

## 4. Discussion

In this meta-analysis, the relationship between these five SNPs in microRNAs (rs2910164, rs11614913, rs3746444, rs11134527, and rs531564) and the risk of cervical cancer was evaluated from 11 published studies. We demonstrated that no evidence was found for the association between miRNA-146a rs2910164, miR-196a2 rs11614913, miRNA-499 rs3746444, and miR-218 rs11134527 and cervical cancer risk in any genetic models. The miR-124 rs531564 polymorphism was associated with a statistically increased risk of cervical cancer in the homozygote model, dominant model, and recessive model. However, due to limited samples and heterogeneity, this finding should be interpreted with caution.

Recent studies have demonstrated that miRNAs are abnormally expressed in cervical cancer and play a role in tumor generation [28]. miR-146a (rs2910164) involves a G>C nucleotide substitution, which changes the G:U pair to C:U [29]. Different locations may cause the function of miR-146a to be different, and the binding affinity to the 3′-UTR region of the target gene may also be different [30, 31]. Furthermore, it is reported that miR-146a can restrict the NF-*κ*B signaling pathway mediated by IRAK1 and TRAF6, which is an important signaling factor involved in the occurrence of cancers [32, 33]. Previous studies have shown that miR-124 is closely related to the occurrence and development of cervical cancer [34, 35]. Evidence has been shown that miR-124 rs531564 polymorphism affects miRNA processing, and the G allele affects the circular secondary structure of pre-miR-124 and changes the expression of other miRNAs [36].

In 2013, Yamamoto et al. found that the expression of miR-218 in cervical cancer tissues was significantly lower than that of adjacent noncancerous tissues. The restoration of miR-218 significantly inhibited the proliferation, migration, and invasion of cancer cells [37]. Laminin-5 *β*3 (LAMB3) has been verified as a transcriptional target of miR-218 [38], and the expression of LAMB3 is increased in the presence of the HPV-16 E6 oncoprotein, and this effect is mediated by miR-218 [19]. The miR-196a2 is located on the human chromosome 12q13.13. In 2016, Torruella-Loran et al. reported that rs11614913 in miR-196a2 has the function of regulating the expression of several genes involved in cancer [39]. Results of a functional study showed that the mutation reduced the efficiency of miRNA precursor processing into its mature form and reduced the ability to regulate target genes [40]. miR-499 is one of the microRNAs that play an important role in posttranscriptional regulation by regulating multiple genes and signal transduction pathways. The miR-499 (rs3746444) polymorphism leads to mismatches from A:U to G:U in the stem structure of the miR-499 precursor. The presence of this mismatch will affect Sox6 and Rod1 genes, which are important for the occurrence of cancers [41, 42].

Significant heterogeneity was found in this meta-analysis for association of miR-218 rs11134527, miR-146a rs2910164, miR-196a2 rs11614913, miR-499 rs3746444, and cervical cancer. Therefore, we conducted a subgroup analysis based on sample size and HWE. When we restricted the sample size to the large sample size group and the HWE group, heterogeneity is significantly reduced for miR-196a2 rs11614913. However, the small sample size group of miR-196a2 includes only two studies, and the large sample size group includes three studies. Therefore, further research is needed to confirm these results. Significant heterogeneity was also found for the association of miRNA-146a rs2910164, miR-499 rs3746444, miR-218 rs11134527, and cervical cancer in the large sample size group and the HWE group. However, when we delete some researches in each analysis, heterogeneity is significantly reduced, suggesting that these researches contributed to the source of heterogeneity. However, owing to the limited articles included, further evaluation of the results is still needed.

In the sensitivity analysis, no significant changes were found after omitting one study at a time, indicating that our meta-analysis results are relatively stable and credible.

Our meta-analysis has several advantages. First, this is the first meta-analysis of the association between five miRNA polymorphisms and the susceptibility to cervical cancer. In addition, according to the methodological quality assessment, all included studies are of high quality. Moreover, there are no restrictions in the literature search, so the selection bias is well controlled. Our meta-analysis has some limitations. Firstly, the number of included studies is too few. Secondly, heterogeneity was detected in miR-146a rs2910164, miR-196a2 rs11614913, miR-499 rs3746444, and miR-218 rs11134527. After deleting some studies in each analysis, heterogeneity is significantly reduced. The pooled ORs became significant without evidence of heterogeneity. Thirdly, the included studies were all Asian ethnicity; therefore, it is uncertain whether these results can be generalized to other populations. Fourth, publication bias might also exist, because only published studies were included in this meta-analysis, and results that are not statistically significant usually have less chance of publication.

## 5. Conclusion

In summary, our results suggested that miR-124 rs531564 polymorphism is significantly associated with increased risk of cervical cancer, while miR-146a rs2910164, miR-196a2 rs11614913, miR-499 rs3746444, and miR-218 rs2292832 polymorphisms may not be associated with the susceptibility of cervical cancer. However, since few studies have been included, there is not enough data to fully confirm the association between cervical cancer and the five miRNA polymorphisms, so the results should be interpreted with caution.

## Figures and Tables

**Table 1:** Characteristics of studies included in the meta-analysis.

Study ID	Year	Country	Ethnicity	Genotyping methods	Sample size (case/control)	Case			Control			*P* for HWE	Quality
miR-146a rs2910164 G>C						CC	CG	GG	CC	CG	GG		
Bin Zhou	2011	China	Asian	PCR-RFLP	266/309	70	113	43	116	159	34	0.060	9
Shizhi Wang	2019	China	Asian	TaqMan	954/1339	318	475	141	471	631	212	0.978	9
Nisha Thakur	2019	India	Asian	PCR-RFLP	150/150	21	49	80	28	49	73	0.001	8
Cong Yue	2011	China	Asian	PCR-RFLP	447/443	105	224	118	150	206	87	0.285	9
Shruti SrivaStava	2017	India	Asian	PCR-RFLP	184/164	18	85	81	8	72	84	0.130	9
Guange Chen	2020	China	Asian	TaqMan	290/445	118	123	49	152	209	80	0.580	9
miR-196a2 rs11614913 T>C						TT	CT	CC	TT	CT	CC		
Bin Zhou	2011	China	Asian	PCR-RFLP	266/309	57	123	46	82	169	58	0.077	9
Shizhi Wang	2019	China	Asian	TaqMan	954/1339	271	464	194	424	629	269	0.201	9
Zhiling Yan	2019	China	Asian	TaqMan	547/567	117	277	153	153	282	132	0.926	9
Nisha Thakur	2019	India	Asian	PCR-RFLP	150/150	17	58	75	57	51	42	0.0001	8
Shruti SrivaStava	2017	India	Asian	PCR-RFLP	184/164	71	93	20	62	81	21	0.492	9
Guange Chen	2020	China	Asian	TaqMan	290/445	105	125	58	140	220	80	0.691	9
miR-499 rs3746444 A>G						AA	AG	GG	AA	AG	GG		
Bin Zhou	2011	China	Asian	PCR-RFLP	266/309	134	84	8	223	71	15	0.005	8
Shizhi Wang	2019	China	Asian	TaqMan	954/1339	675	228	27	946	339	35	0.485	9
Shruti SrivaStava	2017	India	Asian	PCR-RFLP	184/164	26	78	80	54	76	34	0.449	9
Nisha Thakur	2019	India	Asian	PCR-RFLP	150/150	25	47	78	21	49	80	0.005	8
miR-218 rs11134527 A>G						AA	AG	GG	AA	AG	GG		
Ting-Yan Shi	2013	China	Asian	TaqMan	1565/1391	588	752	225	512	638	241	0.083	9
Xiaoyi Zhou	2010	China	Asian	PCR-RFLP	703/713	268	316	101	247	339	127	0.568	9
Li Chuanyin	2017	China	Asian	TaqMan	609/583	233	294	92	242	273	68	0.497	9
Guange Chen	2020	China	Asian	TaqMan	290/445	93	123	61	185	160	85	<0.001	8
miR-124 rs531564 G>C						CC	CG	GG	CC	CG	GG		
Xingdong Xiong	2014	China	Asian	PCR-LDR	208/107	91	15	1	151	51	6	0.507	9
Henghui Wu	2014	China	Asian	PCR-LDR	158/260	134	22	2	184	66	10	0.194	9
Li Chuanyin	2017	China	Asian	TaqMan	609/583	17	144	448	7	118	458	0.846	9

**Table 2:** Summary of pooled ORs in the meta-analysis.

	*N*	OR	*P*_*H*_	OR	*P*_*H*_	OR	*P*_*H*_	OR	*P*_*H*_	OR	*P*_*H*_
miRNA-146a		C/G		GC/GG		CC/GG		GC+CC/GG		CC/GC+GG	
Overall	6	1.08 (0.90-1.30)	0.001	0.94 (0.76-1.15)	0.140	0.85 (0.57-1.26)	0.001	0.90 (0.70-1.15)	0.014	0.90 (0.68-1.19)	0.002
Sample size											
Large	4	1.13 (0.91-1.40)	0.001	0.88 (0.67-1.16)	0.072	0.77 (0.49-1.20)	0.001	0.83 (0.60-1.15)	0.012	0.86 (0.64-1.16)	0.003
Small	2	0.96 (0.59-1.57)	0.038	1.08 (0.78-1.50)	0.390	1.22 (0.37-4.04)	0.029	1.06 (0.67-1.69)	0.132	1.17 (0.40-3.41)	0.043
HWE											
*P* > 0.05	5	1.06 (0.86-1.31)	0.000	0.93 (0.73-1.19)	0.083	0.88 (0.56-1.39)	0.000	0.91 (0.67-1.22)	0.007	0.93 (0.69-1.27)	0.001
*P* < 0.05	1	1.24 (0.88-1.74)	—	0.91 (0.55-1.52)	—	0.68 (0.36-1.31)	—	0.83 (0.53-1.31)	—	0.71 (0.38-1.32)	—
miRNA-196a2		C/T		TC/TT		CC/TT		TC+CC/TT		CC/TC+TT	
Overall	6	0.83 (0.67-1.04)	0.000	1.19 (0.90-1.58)	0.001	1.41 (0.94-2.10)	0.000	1.28 (0.93-1.75)	0.000	1.23 (0.97-1.57)	0.021
Sample size											
Large	4	0.92 (0.84-1.02)	0.284	1.06 (0.86-1.31)	0.106	1.19 (1.00-1.41)	0.369	1.10 (0.90-1.33)	0.112	1.12 (0.97-1.29)	0.676
Small	2	0.62 (0.22-1.78)	0	1.91 (0.52-7.08)	0.001	2.24 (1.32-15.50)	0.000	2.12 (0.44-10.21)	0.000	1.49 (0.49-4.52)	0.006
HWE											
*P* > 0.05	5	0.93 (0.85-1.02)	0.336	1.06 (0.89-1.26)	0.182	1.16 (0.99-1.37)	0.394	1.08 (0.92-1.28)	0.173	1.10 (0.96-1.27)	0.681
*P* < 0.05	1	0.36 (0.26-0.51)	—	3.81 (1.97-7.37)	—	5.99 (3.09-11.59)	—	4.80 (2.62-8.76)	—	2.57 (1.59-4.15)	—
miRNA-499		G/A		AG/AA		GG/AA		AG+GG/AA		GG/AG+AA	
Overall	4	0.76 (0.50-1.15)	0	1.34 (0.82-2.20)	0.001	1.42 (0.61-3.30)	0	1.42 (0.83-2.42)	0	1.27 (0.68-2.35)	0.002
Sample size											
Large	2	0.86 (0.58-1.27)	0.025	1.34 (0.65-2.75)	0.001	1.03 (0.66-1.60)	0.705	1.28 (0.70-2.35)	0.003	0.99 (0.64-1.53)	0.413
Small	2	0.68 (0.27-1.72)	0	1.34 (0.52-3.48)	0.035	2.01 (0.35-11.56)	0	1.58 (0.44-5.63)	0.002	1.67 (0.55-5.05)	0.001
HWE											
*P* > 0.05	2	0.67 (0.28-1.58)	0	1.36 (0.61-3.00)	0.007	2.28 (0.52-9.98)	0	1.64 (0.54-5.01)	0	1.80 (0.69-4.74)	0.006
*P* < 0.05	2	0.86 (0.55-1.36)	0.048	1.33 (0.56-3.16)	0.029	0.84 (0.50-1.43)	0.886	1.26 (0.59-2.70)	0.035	0.89 (0.60-1.34)	0.583
miRNA-218		G/A		AG/AA		GG/AA		AG+GG/AA		GG/AG+AA	
Overall	4	0.98 (0.83-1.15)	0.003	1.07 (0.89-1.29)	0.053	1.01 (0.73-1.40)	0.006	1.06 (0.86-1.31)	0.010	0.97 (0.76-1.23)	0.035
Sample size											
Large	4	0.98 (0.83-1.15)	0.003	1.07 (0.89-1.29)	0.053	1.01 (0.73-1.40)	0.006	1.06 (0.86-1.31)	0.010	0.97 (0.76-1.23)	0.035
Small	0	—	—	—	—	—	—	—	—	—	—
HWE											
*P* > 0.05	3	1.03 (0.88-1.21)	0.019	0.99 (0.88-1.13)	0.318	0.92 (0.66-1.28)	0.021	0.97 (0.82-1.16)	0.091	0.92 (0.69-1.24)	0.031
*P* < 0.05	1	0.79 (0.63-0.98)	—	1.53 (1.09-2.15)	—	1.43 (0.94-2.16)	—	1.49 (1.09-2.05)	—	1.15 (0.79-1.66)	—
miRNA-124		C/G		GC/GG		CC/GG		GC+CC/GG		CC/GC+GG	
Overall	3	0.58 (0.40-0.83)	0.097	1.27 (0.97-1.66)	0.899	2.87 (1.40-5.91)	0.887	1.38 (1.07-1.80)	0.409	2.26 (1.58-3.23)	0.979
Sample size											
Large	2	0.46 (0.32-0.66)	0.858	1.70 (0.47-6.17)	0.967	3.63 (1.04-12.63)	0.996	3.13 (0.90-10.85)	0.995	2.24 (1.51-3.31)	0.860
Small	1	0.75 (0.59-0.95)	—	1.25 (0.95-1.64)	—	2.48 (1.02-6.04)	—	1.32 (1.01-1.72)	—	2.36 (0.97-5.74)	—
HWE											
*P* > 0.05	3	0.58 (0.40-0.83)	0.097	1.27 (0.97-1.66)	0.899	2.87 (1.40-5.91)	0.887	1.38 (1.07-1.80)	0.035	2.26 (1.58-3.23)	0.979
*P* < 0.05	0	—	—	—	—	—	—	—	—	—	—

**Table 3:** Summary of pooled ORs after deleting some articles.

	*N*	OR	*P*_*H*_	OR	*P*_*H*_	OR	*P*_*H*_	OR	*P*_*H*_	OR	*P*_*H*_
miRNA-146a		C/G		GC/GG		CC/GG		GC+CC/GG		CC/GC+GG	
Overall	6	1.08 (0.90-1.30)	0.001	0.94 (0.76-1.15)	0.140	0.85 (0.57-1.26)	0.001	0.90 (0.70-1.15)	0.014	0.90 (0.68-1.19)	0.002
After deleting three articles	3	1.36 (1.19-1.56)	0.800	0.76 (0.60-0.97)	0.379	0.53 (0.40-0.70)	0.683	0.68 (0.54-0.85)	0.406	0.66 (0.53-0.82)	0.638
miRNA-499		G/A		AG/AA		GG/AA		AG+GG/AA		GG/AG+AA	
Overall	4	0.76 (0.50-1.15)	0.000	1.34 (0.82-2.20)	0.001	1.42 (0.61-3.30)	0.000	1.42 (0.83-2.42)	0.000	1.27 (0.68-2.35)	0.002
After deleting two articles	2	1.04 (0.90-1.21)	0.730	0.93 (0.77-1.12)	0.674	0.97 (0.65-1.46)	0.514	0.94 (0.79-1.13)	0.633	1.01 (0.72-1.42)	0.673
miRNA-218		G/A		AG/AA		GG/AA		AG+GG/AA		GG/AG+AA	
Overall	4	0.98 (0.83-1.15)	0.003	1.07 (0.89-1.29)	0.053	1.01 (0.73-1.40)	0.006	1.06 (0.86-1.31)	0.010	0.97 (0.76-1.23)	0.035
After deleting two articles	2	1.11 (1.02-1.21)	0.395	0.97 (0.85-1.10)	0.214	0.79 (0.66-0.94)	0.594	0.92 (0.81-1.04)	0.235	0.80 (0.68-0.94)	0.982

## Data Availability

The data supporting this meta-analysis are from previously reported studies which have been cited.

## Supplementary Materials

Supplementary Materials Table S1: scale for quality assessment.
