# Supplementary material for: The Association between Five Genetic Variants in MicroRNAs (rs2910164, rs11614913, rs3746444, rs11134527, and rs531564) and Cervical Cancer Risk: A Meta-Analysis
Source: Biomed Res Int. 2021 Mar 15;2021:9180874. doi: 10.1155/2021/9180874 (PMC7987420; doi:10.1155/2021/9180874)
Supplement: Supplementary Materials — Table S1: scale for quality assessment. [file 9180874.f1.docx]

Table S1 Scale for quality assessment

| Criteria | Score |
| --- | --- |
| A. Representativeness of subjects  Consecutive/randomly selected from population with clearly defined sampling frame  Consecutive/randomly selected from population without clearly defined sampling frame  Not described | 2  1  0 |
| B. Ascertainment of cervical cancer  Histopathologic confirmation  Diagnosis of cervical cancer by patient medical record  Not described | 2  1  0 |
| C. genotyping methods  Genotyping done under “blind” conditions  Unblinded or not mentioned | 1  0 |
| D. HWE  Hardy–Weinberg equilibrium in control subjects  Hardy–Weinberg disequilibrium in control subjects  Not mentioned | 2  1  0 |
| E. Assessment of association  Assess association between genotypes and cervical cancer with appropriate statistics and adjustment for confounders  Assess association between genotypes and cervical cancer with appropriate statistics without adjustment for confounders  Inappropriate statistics used | 2  1  0 |
| Total | 9 |
